# Acorn: A grid computing system for constraint based modeling and visualization of the genome scale metabolic reaction networks via a web interface

**DOI:** 10.1186/1471-2105-12-196

**Published:** 2011-05-24

**Authors:** Jacek Sroka, Łukasz Bieniasz-Krzywiec, Szymon Gwóźdź, Dariusz Leniowski, Jakub Łącki, Mateusz Markowski, Claudio Avignone-Rossa, Michael E Bushell, Johnjoe McFadden, Andrzej M Kierzek

**Affiliations:** 1 Institute of Informatics University of Warsaw, Poland; 2 Faculty of Health and Medical Sciences, University of Surrey, Guildford, GU2 7XH, UK

## Abstract

**Background:**

Constraint-based approaches facilitate the prediction of cellular metabolic capabilities, based, in turn on predictions of the repertoire of enzymes encoded in the genome. Recently, genome annotations have been used to reconstruct genome scale metabolic reaction networks for numerous species, including *Homo sapiens*, which allow simulations that provide valuable insights into topics, including predictions of gene essentiality of pathogens, interpretation of genetic polymorphism in metabolic disease syndromes and suggestions for novel approaches to microbial metabolic engineering. These constraint-based simulations are being integrated with the functional genomics portals, an activity that requires efficient implementation of the constraint-based simulations in the web-based environment.

**Results:**

Here, we present Acorn, an open source (GNU GPL) grid computing system for constraint-based simulations of genome scale metabolic reaction networks within an interactive web environment. The grid-based architecture allows efficient execution of computationally intensive, iterative protocols such as Flux Variability Analysis, which can be readily scaled up as the numbers of models (and users) increase. The web interface uses AJAX, which facilitates efficient model browsing and other search functions, and intuitive implementation of appropriate simulation conditions. Research groups can install Acorn locally and create user accounts. Users can also import models in the familiar SBML format and link reaction formulas to major functional genomics portals of choice. Selected models and simulation results can be shared between different users and made publically available. Users can construct pathway map layouts and import them into the server using a desktop editor integrated within the system. Pathway maps are then used to visualise numerical results within the web environment. To illustrate these features we have deployed Acorn and created a web server allowing constraint based simulations of the genome scale metabolic reaction networks of *E. coli*, *S. cerevisiae *and *M. tuberculosis*.

**Conclusions:**

Acorn is a free software package, which can be installed by research groups to create a web based environment for computer simulations of genome scale metabolic reaction networks. It facilitates shared access to models and creation of publicly available constraint based modelling resources.

## Background

Analysis of biochemical reaction networks, to enable predictions about the phenotype from a catalogue of molecular parts encoded in the genome, fulfils a major objective of Systems Biology. Due to the scarcity of detailed quantitative information about individual enzyme rate constants and intracellular concentrations, genome scale models have, so far, only been constructed for the special case of the metabolic reaction networks operating under steady state conditions. These models capitalize on the fact that metabolic conversions occur in the cell with very high rates and therefore, for the given set of genes active under experimental conditions of interest, the network of metabolic reactions can be considered to be in a quasi-steady state (where metabolite concentrations do not change). The system is then modelled by a set of linear relationships between the calculated reaction fluxes. Although, the linear system corresponding to genome scale models is underdetermined it can be exploited by Linear Programming (LP) based optimization of the objective function that represents the metabolic capability of interest. The objective function is defined as the flux through a specific reaction or the flux towards a specific metabolite. LP optimization results in a unique value for the objective function, but the corresponding flux distribution is not unique. The range of each individual reaction flux in all flux distributions that are consistent with the maximal objective function value can be exploited by the iterative LP protocols, described in the following sections. This approach is referred to as constraint based [1], because it evaluates metabolic capabilities under the set of constraints expressed by balance equations, known quantitative values of certain fluxes (determined by *in vitro *experimentation) and information (from the literature) about maximal capacity and reversibility of the reactions. The acquisition of this information can be initiared by analysis of the genome sequence of interest, and this approach has been applied to the reconstruction of genome scale metabolic reaction networks (GSMNs) for major model organisms [2-5], pathogens [6-8] and microorganisms used in industrial bioprocesses [9,10]. The first reconstruction of human metabolism is also available [11] and is currently being used to investigate mechanisms of metabolic diseases [12,13].

The availability of full genome sequences has motivated the development of functional genomics internet portals, where a multiplicity of sequence-derived, high-throughput and traditional experimental data are integrated through appropriate comparative analysis protocols. The integration of constraint-based simulations of GSMNs with these portals will equip users, for the first time, with tools that will enable predictions of the metabolic phenotype of cells under defined experimental conditions, determined in terms of a set of nutrients present in the medium.

The concept of gene essentiality provides a good illustration of these new capabilities. Currently, functional genomics portals describe each gene in terms of comparison to similar sequences in other organisms, direct experimental evidence about the function under particular experimental conditions and high throughput datasets in which the expression of the gene has been observed. A constraint-based analysis of the appropriate GSMN, integrated with this system, would allow this content to be dependent on experimental conditions, specified by the user. The user could specify the composition of the growth medium and then compute the function of the gene expressed as a set of metabolic capabilities for which this gene is essential under user-defined experimental conditions.

So far, there have been the following attempts at creating web-based resources, allowing computation with GSMN models with subsequent linking of models and results to functional genomics resources. The GSMN-TB [6] is the first reconstruction of the GSMN of *Mycobacterium tuberculosis*, the causative agent of Tuberculosis. In this case, the web interface allows the specification of the growth medium composition, and using this information, provides a calculation of the maximal theoretical flux towards biomass as well as a gene essentiality prediction. It also implements a reaction essentiality scan and Flux Variability Analysis (FVA) [14], which are iterative protocols performing respectively one or two linear programming optimizations for each reaction in the model. All of the gene names in the model are linked to Tuberculist [15], a functional genomics portal dedicated to Mycobacteria. The CycSim [16] system allows the computation of the maximal theoretical flux towards selected metabolites and does not implement the iterative protocols described above. However, it implements a sophisticated user interface, enabling the specification of experimental conditions and perturbations. It also allows visualisation of numerical data on pathway maps imported from KEGG [17] or PathwayTools [18] software. The WEbcoli [19] server allows modification and constraint based simulation of the *Escherichia coli *iJR904 model. The server displays metabolic pathway maps and overlays simulation results on these diagrams. Only a single evaluation of the objective function is provided and no iterative simulation approaches are available. We also note that none of the web servers described above are available as software that can be downloaded and used to create new web servers at the users institution. Recently, the BioMet Toolbox [20] server has made as set of constraint-based modelling tools developed by Nielsen's group available via web interface. The advantage of BioMet Toolbox is a wide range of methods that can be run. However, in comparison with the servers mentioned above it is not integrated with functional genomics portals and visualisation tools. The user is expected to create text file in the format of particular tool or download the file from repository of models and subsequently upload this file to the form of the web interface of the particular tool. Other servers have integrated databases of models and format the models for presentation via web interface. Reactions are linked through gene names to functional genomics portals, thus facilitating interpretation of models in the context of genome annotation. The results are also formatted for web presentation and numerical data are visualised on network maps.

In this work we present Acorn, a grid computing system, which can be deployed at the users institution and used to create web based resources for constraint based simulations of GSMN models. Research groups can install Acorn, set up user accounts and import models in the SBML format [21]. Users can also define *in silico *experiments by specifying nutritional conditions and run virtual experiments in order to calculate the maximum theoretical value of the objective function, identify essential reactions, predict gene essentiality and perform Flux Variability Analysis (FVA) [14]. Models and experiments can be shared between users. Our grid-based Acorn architecture facilitates the implementation of computationally intensive, iterative protocols such as FVA within interactive web environments and can be easily scaled up as the number of users and models grow. The web server part of the system allows the visualization of the numerical results on pathway maps defined by the user. Acorn also includes an integrated graphic editor for fast creation of metabolic map layouts and a command line tool, which can be used in connection with other interfaces. To demonstrate these features we have used Acorn to implement a web resource providing constraint-based simulations of the GSMNs of *Saccharomyces cerevisiae, E. coli *and *M. tuberculosis *:

http://sysbio3.fhms.surrey.ac.uk:8080/acorn/homepage.jsf.

## Implementation

In this section we describe the implementation details of Acorn. We also explain and justify the technological and architectural choices made, and the design approaches applied to the implementation of the system.

**Figure 1:**
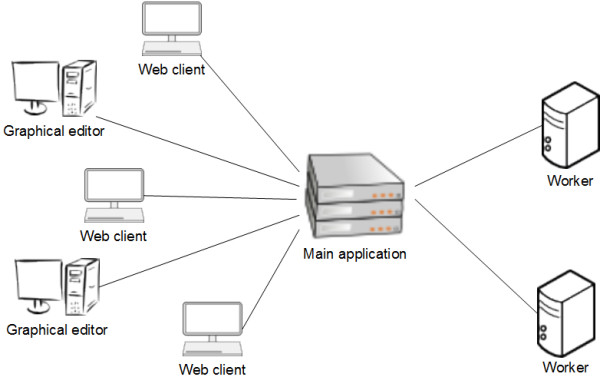
**Components of the Acorn system**.

The Acorn system consists of three components (Figure 1):

1. The main application, which includes a web based user interface for defining and executing the required computational task as well a database for storing models, experiments and experimental results,

2. The worker console application for performing the computations requested by users via the web interface,

3. A graphical editor desktop application for defining which parts of the model are to be visualized and how.

The user interface of the main application is web based, designed with ergonomics in mind and optimised for handling of large models. As an example of this latter feature, the user can quickly filter long lists with an AJAX (Asynchronous JavaScript and XML) based mechanism, i.e., without the delays that might be incurred in transferring data between the browser and the web server. An example of the ergonomic approach is the positioning of key control buttons such that they are always visible in the same place on the screen using specially defined CSS (Cascading Style Sheets). This facilitates working with long lists. A useful outcome of this approach is that the user does not have to scroll to start the simulation after accepting the choice of optimisation target on the reaction list.

**Figure 2:**
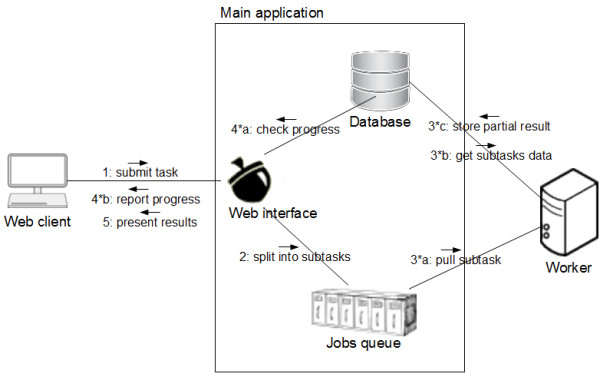
**Interaction between the worker, main application and web client**.

Since computations with Acorn can be time intensive, we have chosen an architecture (Figure 2) that allows for easy distribution of the workload between many machines, enabling the rapid and easy creation of a computational cluster. Computations commissioned by the users are automatically divided into subtasks, which can be independently computed by workers running on the same or different machines. The main application places subtask specifications in a queue to be pulled by one of the workers. Each worker cyclically consumes the current oldest subtask from the queue, accesses its corresponding model data in the main applications' database, performs the computation and stores results back in the database. In addition to submission date, the less time consuming tasks are given higher priority. Then, if the queue contains more subtasks, the worker pulls the next one or, if no more subtasks are currently available, waits until a new task appears. The use of the queue based pull model where processing jobs are pulled by free workers as opposed to being pushed out by the main application results in an optimal workload distribution. It also renders administration of the system easier, because the content of the queue can be monitored and workers can be added or removed according to needs and availability of free processing power.

The system is implemented in Java with the use of the Java Enterprise Edition (Java EE) technology stack. The main application can be deployed on a Java EE 5.0 compatible application server as the Open Source GlassFish 2.1 https://glassfish.dev.java.net/. Acorn profits from the maturity and enterprise features of Java EE component technologies. For example, operations on the queue and database are subject to transaction control, so the computations can be consistently completed in situations like concurrent execution of many workers, failure of an active worker or restart of the main application. The GlassFish server is also equipped with necessary tools for monitoring and tuning. Furthermore, the flexibility of the enterprise programming environment allows the deployment of Acorn with any relational database management system providing JDBC drivers. The current version has been tested with Java Derby http://db.apache.org/derby/ and MySQL http://www.mysql.com databases.

Linear programming calculations are performed using the appropriate functions of the GLPK library http://www.gnu.org/software/glpk/. The linear programming engine is a binary executable file implemented in C++. The worker starts the linear programming engine using the operating system interface of Java, which sends the linear programming problem to its standard input and retrieves results from standard output without creating any temporary files. The engine is also available for users as a standalone command line tool, which can be used to implement other interfaces.

The graphical editor is a standalone Java desktop application built on the NetBeans Platform, which is a generic framework for Swing applications http://platform.netbeans.org/. It runs on all platforms where the Java runtime environment is available. It accesses the main application to obtain the model data and to store defined visualisations for presentation in the web interface. This communication is performed though a Web Service provided by the main application, which, for the user, has the added advantage that it will not be blocked by firewalls.

## Results

Distribution of Acorn can be implemented by downloading from Google Code repository http://code.google.com/p/a-c-o-r-n/. The main application can be installed on any platform running Java VM, the Glassfish application server and a relational database management system. The binaries of the linear programming engine are available for Linux, MacOS × and Windows operating systems and, together with the Java VM, are necessary to run the worker. The graphical editor desktop application can be used on any system running Java VM. The current version of Acorn provides the following features.

### A web-based user interface to GSMN models

**Figure 3:**
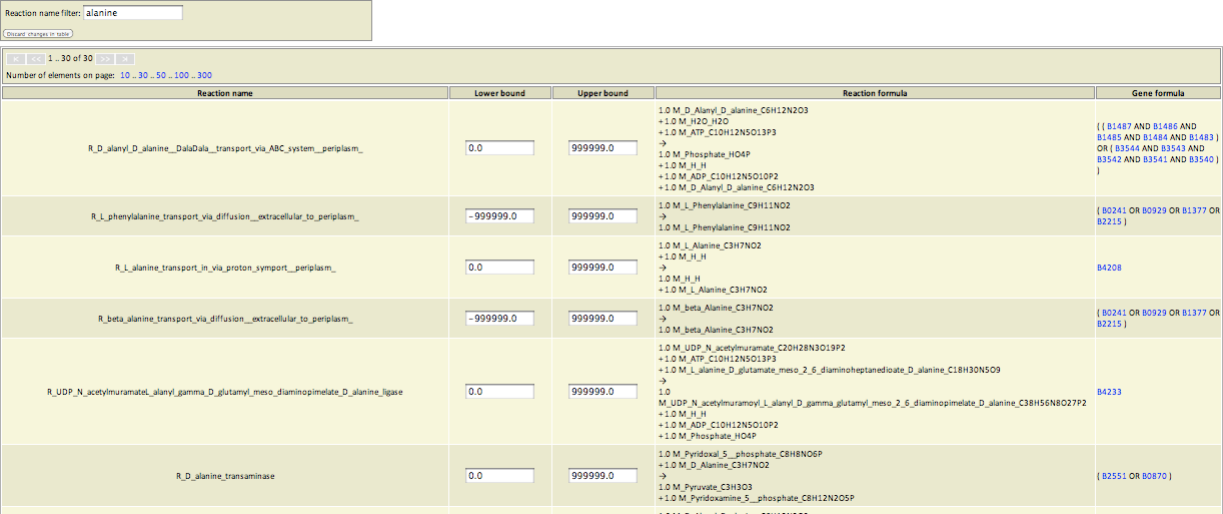
**The web page representingt GSMN**. The table containing reaction names has been interactively filtered for keyword "alanine" in the reaction name. Reaction bounds are editable. The gene names in the Boolean gene-reaction association formula are linked to the functional genomics portal chosen by the user who created the model.

The user logs into the system via web interface and can browse GSMN models in tabular format (Figure 3). The rows of reaction tables contain reaction name, reaction formula and lower and upper flux bounds. The last cell of the reaction entry contains gene-reaction association formula representing genes encoding protein subunits of enzymes catalysing this reaction. The genes encoding components of multisubunit enzymes are linked with AND boolean statement and paralogous genes are represented by OR statements. Gene names are linked to gene pages of selected functional genomics resources integrating GSMN model with literature annotations and comparative sequence analysis results. Reaction bounds are editable and are used to set initial conditions of the constraint-based simulations.

### A web interface to four constraint-based analysis protocols

The following computational methods are available:

i) Flux Balance Analysis (FBA) - calculation of the maximal theoretical value of the objective function under nutritional conditions specified by the user;

iI) Gene essentiality prediction - the fluxes through all reactions which require the functionality of a selected gene are constrained to 0 and the maximal theoretical value of the objective function is calculated. If the objective function value equals 0, the gene is declared to be essential for particular metabolic function;

iii) Reaction essentiality scan - each reaction, in turn, is inactivated and the FBA optimisation is run. The result lists the maximal value of the objective function for each single reaction knock-out;

iv) Flux Variability Analysis (FVA) [14] - the flux corresponding to objective function is constrained to its maximal value and the flux through each reaction, in turn, is maximised and minimised. This results in the calculation of the flux range consistent with the maximal value of the objective function. The flux ranges are unique and, unlike flux values obtained from particular single FBA solution, can be used to reason about internal flux distribution under nutritional conditions of interest [22].

To run a simulation via the Acorn web interface the user first selects one of the methods and then specifies the objective function and nutritional conditions. The objective function can be specified by a metabolite name (maximal flux towards metabolite) or reaction name (maximal flux through the reaction). Nutritional conditions are defined by constraining lower and upper bounds of transport reaction fluxes. The Acorn interface allows the specification of bounds for all reactions in the system, allowing for more complex analysis scenarios such as incorporation of internal metabolic flux measurements or simulations of multiple gene knockouts. The keyword searches can be used to quickly find reactions and metabolites of interest. Simulations are scheduled and the user can either wait for the completion of computational tasks, or log out and return to the system later to examine results. The simulation scenario, together with the simulation results are saved into the database as an *in silico *experiment. Users can share *in silico *experiments and visualise their results on pathway diagrams.

### A grid-based environment for distributing computational load

A single execution of FBA is very computationally efficient. For example, calculation of the maximal growth rate for yeast GSMN model iND750 takes 0.73s on 2.13 GHz Intel Core 2 Duo processor CPU when calculated with the linear programming engine of the Acorn system. However, iterative protocols of reaction essentiality scans and FVA require one or two (respectively) evaluations of the objective function for each reaction in the system. Therefore, execution of FVA for the yeast GSMN model containing 1265 reactions takes about 15 minutes of CPU time. This computational time can be substantial for web-based applications, especially if multiple users are simultaneously using the server. The Acorn server, on the other hand, is using grid-based solutions to distribute computational load between multiple computers. The worker program is installed on each computer in the cluster. The worker connects to the Acorn server and receives a set of FBA objective function evaluation tasks to execute. Results of these tasks are sent back to the server (see Implementation section for more details). Therefore, FBA calculations from the Acorn server can be distributed over an arbitrary number of computers in the local area network or the internet, rendering the system scalable to large number of users and computational tasks.

### SBML file import

**Figure 4:**
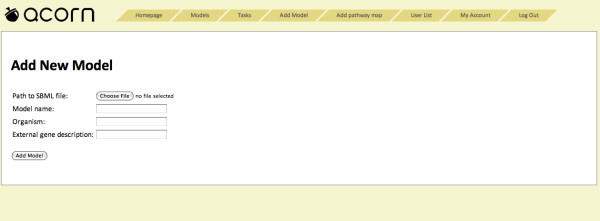
**The model import interface**. The user is prompted to provide the name of the SBML file with the BiGG database annotations representing FBA specific information. Subsequently, the user specifies model name and an organism. The user can than link gene names to the functional genomics portal of choice by providing the HTTP address with the gene name replaced by "%s" string. This address template is then used to link individual genes on model pages to the corresponding pages of the functional genomics portal.

The user can import GSMN models in the SBML format (Figure 4). The reaction capacity bounds and gene-reaction association formulas, features specific to FBA simulations and not implemented in Acorn yet, have to be provided as annotations following BiGG database conventions [23]. The user can upload the file using the web interface. Optionally, the user can input the HTTP address string, which will be concatenated with gene names to create links to the gene pages in the functional genomics portal of the user's choice. The SBML file is parsed and stored in the relational database backend area of the Acorn system.

### A user management, model and in silico experiment sharing

Every new user of Acorn has to register via the web interface. The first user registered, after the software installation step, is given administration privileges. The models are created during SBML file upload, and initially can be only used by the user who uploaded the SBML file. Likewise, *in silico *experiments with a particular model initially belong the user who created the model. The user may chose to share models and *in silico *experiments with other users in the system. This allows for the creation of online communities where users share models and results of numerical simulations. The models and experiments can be also made available to a publicly accessible account, which enables creation of web based resources for constraint based simulations of GSMN models.

### Desktop metabolic pathway layout editor

**Figure 5:**
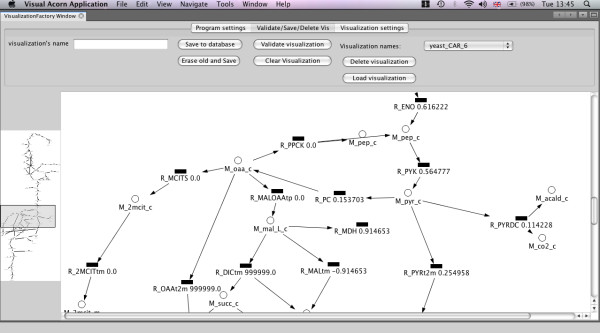
**Screenshot of desktop editor session with *S. cerevisiae *iND750 model**. The user can create the network map where circles represent substances and reactions are represented by rectangles. The symbols are linked to substances and reactions in the GSMN. Therefore, while the map does not need to represent a whole genome scale network, numerical results, which are mapped on the diagram are obtained by the computations with entire model. The map layout is send to the Acorn server and stored in the database to be used for generation of visualisations on the web interface. Numerical results can be also visualised within desktop editor. The figure shows results of the FBA optimisation of biomass flux.

Acorn provides a desktop editor for metabolic pathway diagrams (Figure 5). Diagrams are created in Petri net [24] notation with reactions and substances represented by different symbols. The editor connects to the Acorn database, using an ID and password of one of the Acorn users and prompts this user to select one of the GSMN models available on the user's Acorn account. Subsequently, the user creates the diagram in which every substance and reaction symbol is associated with the substance or reaction in the GSMN. The editor can find all substances (reactions) associated with a given reaction (substance) and then place them on the map. Therefore, after placing the first symbol on the diagram, the user does not have to check subsequent metabolite names and can quickly build a network map by simply following the connections in the GSMN. The diagram is sent to the server and saved in the database.

### A means of visualisation of numerical results on metabolic pathway diagrams

**Figure 6:**
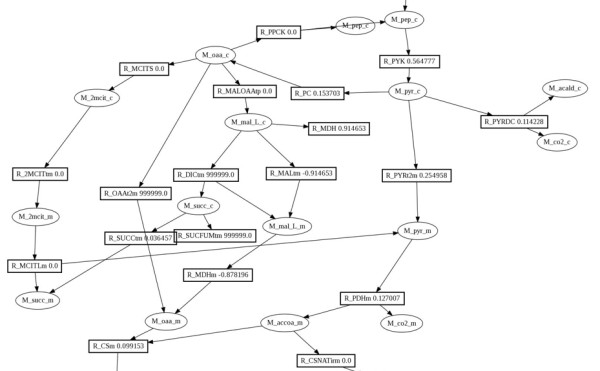
**Visualisation of numerical results on pathway maps within the web interface of Acorn system**. Network map created with desktop editor and stored in the database is used. The reaction fluxes or FVA flux ranges are printed within reaction symbols. The figure shows an image generated by web interface showing reaction fluxes within the part of the central metabolism map of *S. cerevisiae *iND750 model. The results have been obtained during FBA optimisation of biomass flux.

Numerical results of FBA and FVA calculations can be visualised on pathway diagrams (Figure 6). The pathway diagrams created by the user are available within the web system and can be associated with simulation results. Subsequently, the user may create image files where numerical simulation results, representing the fluxes or flux ranges, are printed within the boxes corresponding to the reactions. Numerical results can be also visualised in the same way within the stand-alone pathway editor.

### A stand alone command line tool

The computational engine of the Acorn software is available as a stand-alone tool. The tool can be used to evaluate the objective function of the GSMN model written in simple text file format. The command line tool interprets boolean gene-reaction association formulas and can be therefore used to perform evaluate the objective function for a single gene knockout version of the model. Availability of the Acorn computational engine in the form of a command line tool binary is useful for the users who intend to develop their own interfaces. The tool could be easily integrated with the script languages used by the bioinformatics community and then become a part of an annotation pipeline. It can be also used to create graphics user interfaces other than Acorn. Finally, users of the Acorn web server can also use the command line tool to run some of their computations on their local machines if the resources of the web server are limited or constrained.

### The GSMN web resource

To demonstrate the features of Acorn, we have constructed a web resource for constraint-based simulation of the GSMNs of interest to our research group. The resource contains the GSMN-TB model of *M. tuberculosis *[6], reconstructed by our group and two reconstructions published by other groups: *E. coli *iAF1260 [2] and *S. cerevisiae *iND750 [3]. The iAF1260 and iND750 have been directly uploaded into Acorn from the SBML files available in the BiGG database. This shows that the Acorn model upload tool is compatible with the SBML specification and constraint-based specific annotations used by the COBRA toolbox software and the BiGG database [23]. In the case of GSMN-TB and iND750 models, the flat files accompanying the publications were first converted to an annotated SBML file and subsequently uploaded into the server. The models have been uploaded and tested by one of the users of the system with upload privileges and subsequently published, by sharing them with the "guest" user account available to the public.

Each of the models available within the web resource is linked to the relevant functional genomics portal. The gene names in GSMN-TB model are linked to the Tuberculist database, the *E. coli *model is linked to KEGG database, and the *S. cerevisiae *model is linked to the SGD database. These implementations have demonstrated that the model upload mechanism of Acorn can be used to link reaction lists and results files of GSMN models to major functional genomics resources, thus facilitating interpretation of computer simulations in the context of the wealth of knowledge and comparative analysis results available within these knowledge bases.

The user can use our web resource to run all simulation protocols implemented in Acorn on the models mentioned above. To demonstrate the visualisation functionality of Acorn, we have used the desktop editor to create a layout of glycolytic pathway in Yeast and uploaded this layout into the iND750 model. Users can now visualise fluxes calculated by computer simulation on the pathway layouts of their choice. The fluxes are obtained by the calculations involving all reactions of the entire GSMN, but only the reactions of interest to the users are displayed in a format, which is the most informative for them.

## Conclusions

In this paper, we present the Acorn software package, which can be deployed at the user's institution and used to create web-based resources for constraint based simulations of GSMNs. Our software can be used to make a collection of GSMNs available to a community of users who hold accounts within the system. Users can upload models and run constraint-based simulation methods to make predictions about the metabolic capabilities of organisms under investigation. The models and results are linked to the functional genomic resources through the gene names. Therefore, the results of constraint based calculations can be examined in the context of genome sequences, annotations and sequence similarity-based predictions available in these portals. The grid-based architecture of Acorn allows for the implementation of computationally expensive simulation protocols requiring the execution of thousands of linear programming evaluations for the entire model. Users can visualise numerical results of simulation methods on pathway maps loaded into the system. These maps can represent tthe users view, a part of the model of specific interest to the user concerned, while numerical data visualised on the map are calculated by constraint-based modelling of the entire network. Pathway diagrams are constructed with the desktop tool, which supports rapid assignment of the graphical symbols to substances and reactions present in the model.

One of the most important features of Acorn is user management and model sharing. Select users can upload starting models and share them with others. All other users can create variations of those starting models by modifying their parameters and set their own simulations without affecting the models and simulations run by other users. They may then choose to render the models, simulation scenarios and results visible to other users in the system and, through the guest user account, available to the research community. Therefore, Acorn can be used to create research community websites dedicated to the annotation and investigation of metabolic reaction network models.

In addition to the software that can be used to create a constraint-based modelling server, we also provide a web resource giving access to the GSMN models of *S. cerevisiae*, *E. coli*, and *M. tuberculosis*. Users can either use a guest account or register and gain access to these models without the need of installing and learning modelling environments such as, for example, MATLAB based COBRA toolkits. Users can also use our server to upload their models, if they are represented in the SBML format, and use computational tools implemented within the system to study those models (and share the results with other users).

We believe that Acorn provides several advantages and alternative solutions compared to existing web servers for constrained-based modelling of metabolic networks. It provides advantages when compared to the GSMN-TB server with respect to visualisation and implements a more convenient interface for model specification. Its main advantage over CycSim and WEbcoli is the implementation of the FVA and reaction essentiality scans. FVA is currently the only method, which allows the exploration of internal flux distribution in GSMN.s It is efficiently implemented in Acorn, due to its grid based architecture. Acorn also provides an alternative mechanism for visualising metabolic pathway maps. While CyCsim uses standard KEGG and BioCyc maps, the Acorn server allows the individual user to create their own views, which are linked with the model. Another major advantage of the Acorn environment over CycSim and WEbcoli is that it provides source code distribution, which can be used to create collaborative model development environments and public web resources in other laboratories.

To summarise, we believe that the Acorn server will be very useful for research groups reconstructing and studying GSMNs. Its application will result in establishment of online communities sharing models and results of *in silico *experiments. This will facilitate improvement of metabolic models used in the identification of novel drug targets in microbial pathogens and metabolic engineering of industrially valuable microbial strains.

## Availability and Requirements

Project name: Acorn

Project home page: http://code.google.com/p/a-c-o-r-n/

Surrey GSMN server: http://sysbio3.fhms.surrey.ac.uk:8080/acorn/homepage.jsf

Operating systems(s): Platform independent

Programming language: Java, C++

Other requirements: Java 6.0 or higher, GlassFish Java EE Server v2 or higher

License: GNU GPL v2

Any restrictions to use by non-academics: The use of constraint based modelling results obtained by using iAF1260 and iND750 models on Surrey FBA resource is subjected to the BiGG database license.

## Authors' contributions

AMK and JS designed software functionality. JS designed software architecture, supervised software development and participated in software implementation. LBK, SG, DL, JL implemented database and worker modules. MM implemented pathway visualisation. JL implemented command line computational engine. CAR developed case studies for visualisation of yeast pathways. MB and JMF tested software functionality and participated in writing of the manuscript. AMK and JS wrote the manuscript. All authors read and approved the final manuscript.
